# Supplementary figures and images for: Endothelial activation and stress index in risk stratification and treatment optimization for critically ill patients with acute kidney injury: A retrospective cohort study from MIMIC-Ⅳ database
Source: PLoS One. 2026 May 6;21(5):e0348678. doi: 10.1371/journal.pone.0348678 (PMC13148700; doi:10.1371/journal.pone.0348678)

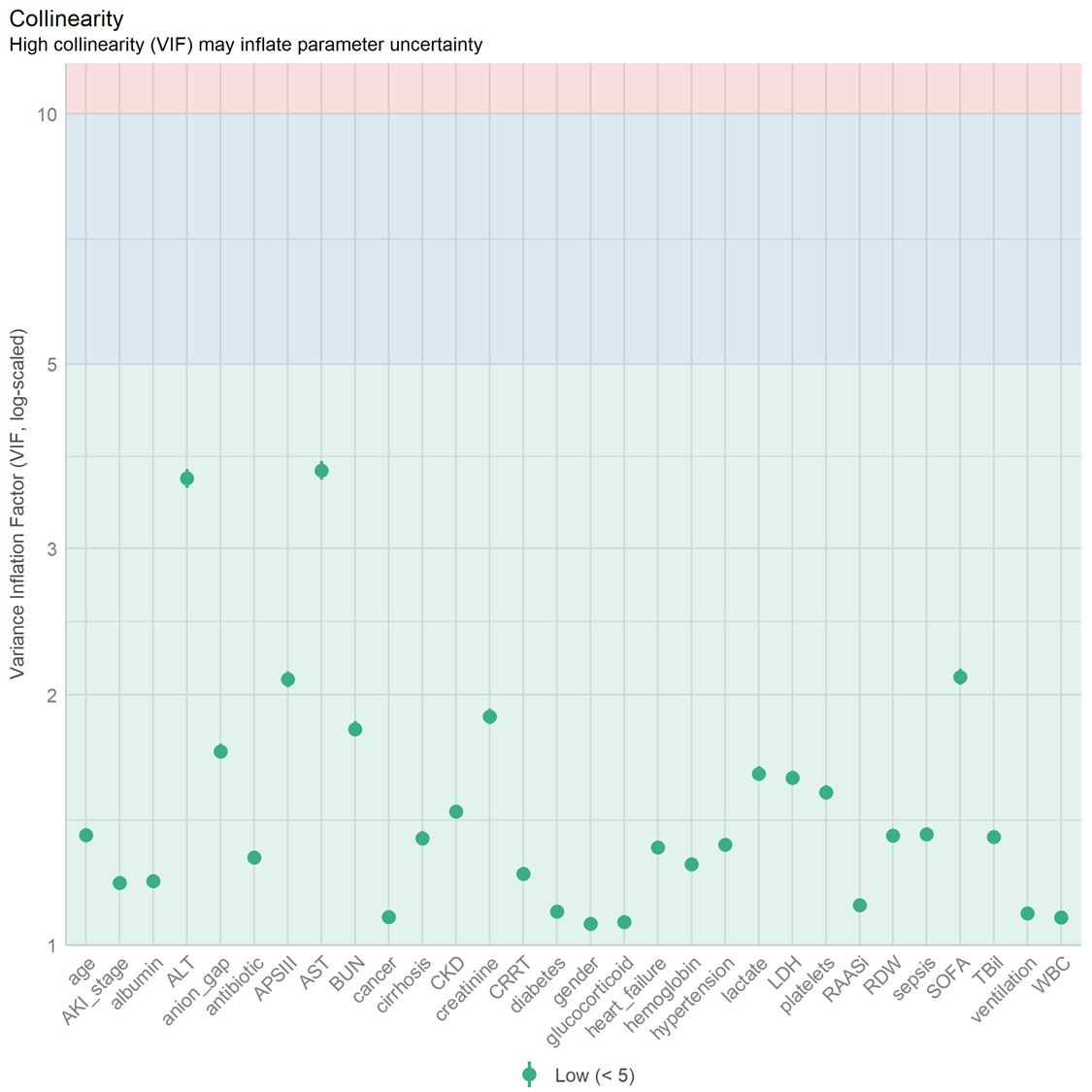

Supplement: S1 Fig — All the values of the variance inflation factors were below 5, and the existence of multicollinearity was not considered. (TIF) [file pone.0348678.s001.tif]

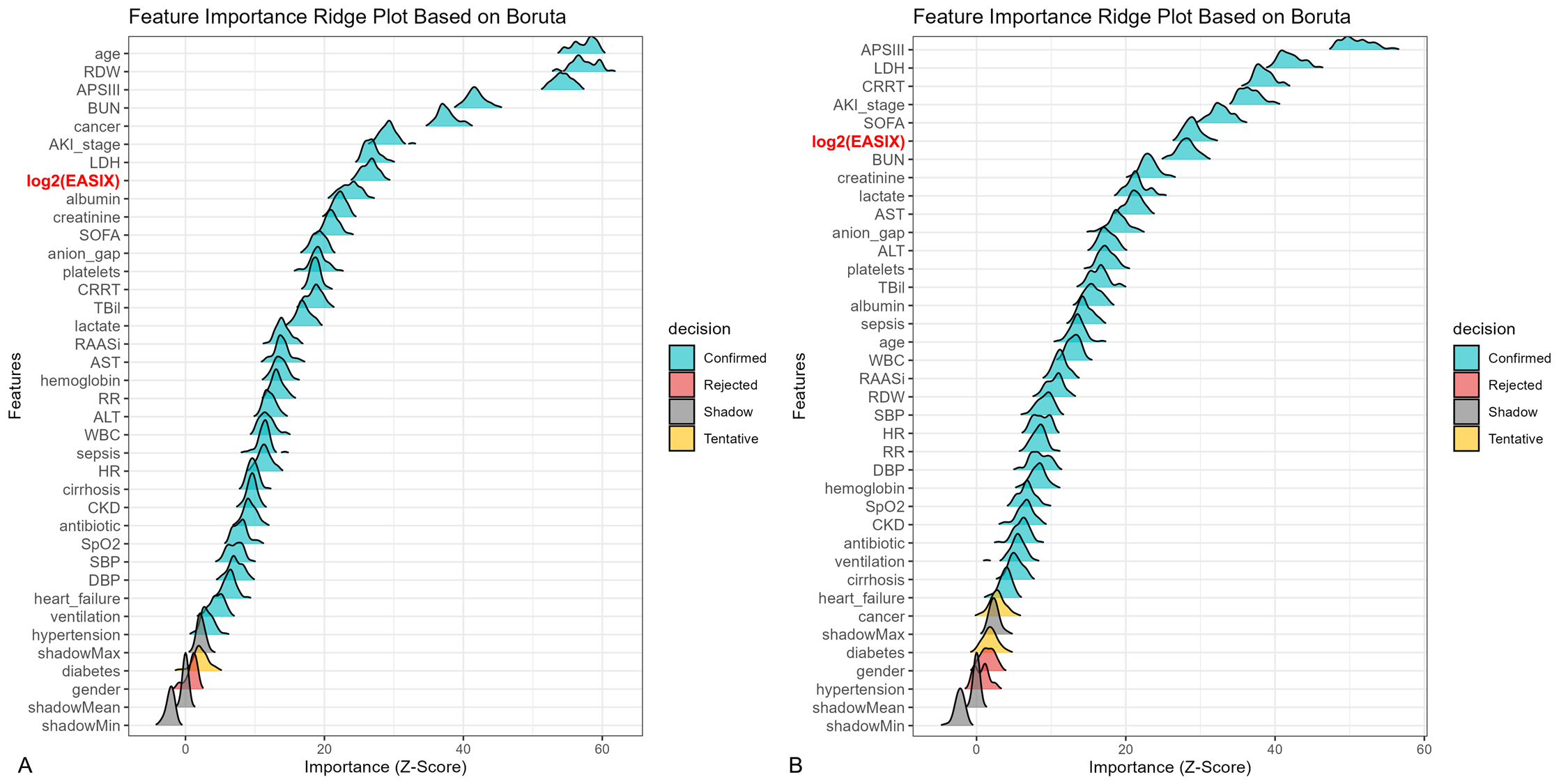

Supplement: S2 Fig — The vertical axis was the name of each variable, and the horizontal axis is the Z value of each variable. The plot showed the Z value of each variable during model calculation. (TIF) [file pone.0348678.s002.tif]
