# Supplementary material for: Endothelial activation and stress index in risk stratification and treatment optimization for critically ill patients with acute kidney injury: A retrospective cohort study from MIMIC-Ⅳ database
Source: PLoS One. 2026 May 6;21(5):e0348678. doi: 10.1371/journal.pone.0348678 (PMC13148700; doi:10.1371/journal.pone.0348678)
Supplement: S1 Table — (DOCX) [file pone.0348678.s003.docx]

**S1 Table. The association between glucocorticoid use and all-cause mortality.**

| **glucocorticoid** | **number** | **model 0** | **model 1** | **model 2** | **model 3** | ***p* for interaction** |
| --- | --- | --- | --- | --- | --- | --- |
| 17624 | | **Hazard Ratio for 1-year mortality (Cox regression)** | | | | **-** |
| No | 12895 | reference | reference | reference | reference |  |
| Yes | 4729 | 1.54 (1.47, 1.61) | 1.47 (1.40, 1.54) | 1.29 (1.23, 1.36) | 1.27 (1.21, 1.34) |  |
| 11346 | | Only in patients with low-level log2(EASIX) | | | | < 0.001 |
| No | 8635 | reference | reference | reference | reference |  |
| Yes | 2711 | 1.57 (1.47, 1.67) | 1.54 (1.45, 1.65) | 1.42 (1.33, 1.52) | 1.42 (1.32, 1.51) |  |
| 6278 | | Only in patients with high-level log2(EASIX) | | | |  |
| No | 4260 | reference | reference | reference | reference |  |
| Yes | 2018 | 1.34 (1.25, 1.44) | 1.32 (1.23, 1.42) | 1.12 (1.05, 1.21) | 1.10 (1.02, 1.18) |  |
| 17624 | | **Relative Risk for ICU mortality (modified Poisson regression)** | | | | **-** |
| No | 12895 | reference | reference | reference | reference |  |
| Yes | 4729 | 1.88 (1.77, 1.99) | 1.65 (1.56, 1.75) | 1.42 (1.34, 1.51) | 1.39 (1.31, 1.47) |  |
| 11346 | | Only in patients with low-level log2(EASIX) | | | | < 0.001 |
| No | 8635 | reference | reference | reference | reference |  |
| Yes | 2711 | 1.92 (1.75, 2.11) | 1.73 (1.58, 1.90) | 1.50 (1.37, 1.65) | 1.48 (1.34, 1.62) |  |
| 6278 | | Only in patients with high-level log2(EASIX) | | | |  |
| No | 4260 | reference | reference | reference | reference |  |
| Yes | 2018 | 1.62 (1.50, 1.74) | 1.50 (1.39, 1.61) | 1.30 (1.21, 1.40) | 1.28 (1.18, 1.37) |  |

model 0: only glucocorticoid use was included.

model 1: age, gender, diabetes, hypertension, AKI stage, CKD, heart failure, cirrhosis, sepsis, and cancer were adjusted.

model 2: age, gender, diabetes, hypertension, AKI stage, CKD, heart failure, cirrhosis, sepsis, and cancer, as well as WBC, hemoglobin, RDW, platelets, AST, ALT, TBil, albumin, creatinine, BUN, LDH, lactate, anion gap, antibiotic, RAASi, ventilation, and CRRT were adjusted.

model 3: age, gender, diabetes, hypertension, AKI stage, CKD, heart failure, cirrhosis, sepsis, cancer, WBC, hemoglobin, RDW, platelets, AST, ALT, TBil, albumin, creatinine, BUN, LDH, lactate, anion gap, antibiotic, RAASi, ventilation, and CRRT, as well as SOFA and APS Ⅲ were adjusted.
