# Supplementary material for: Endothelial activation and stress index in risk stratification and treatment optimization for critically ill patients with acute kidney injury: A retrospective cohort study from MIMIC-Ⅳ database
Source: PLoS One. 2026 May 6;21(5):e0348678. doi: 10.1371/journal.pone.0348678 (PMC13148700; doi:10.1371/journal.pone.0348678)
Supplement: Attachment — Submitted filename: Response to Reviewers.docx [file pone.0348678.s005.docx]

**Dear Editor and Reviewers,**

Many thanks to you for your valuable advice and recognition of our manuscript titled "Endothelial activation and stress index in risk stratification and treatment optimization for critically ill patients with acute kidney injury: A retrospective cohort study from MIMIC-IV database" (Submission ID: PONE-D-25-27246). We sincerely appreciate the time and expertise you have invested in reviewing our work, as your insightful comments have provided not only essential guidance for revising the paper but also important direction for our research. We have carefully studied each comment and made targeted corrections that we believe address your concerns. Specific locations of modifications are highlighted in Red in the revised manuscript, and the comments and suggestions point by point are detailed in the attached Response document.

We look forward to your positive response to the revised work submitted here. *PLoS One* is an influential journal which aims to improve our understanding of diseases. From the papers published in your journal, we have been learning a lot. Hopefully, we could have our article been considered of publication in your journal. Should there been any other corrections we could make, please feel free to contact us.

Sincerely,

Kai Wang

Department of Cardiology, The Second Affiliated Hospital of Chongqing Medical University, Chongqing, China.

Email: nkuwangkai@163.com.

**Editor's comments**

**Comment 1**: Please include the following items when submitting your revised manuscript:

- A letter that responds to each point raised by the academic editor and reviewer(s). You should upload this letter as a separate file labeled 'Response to Reviewers'.
- A marked-up copy of your manuscript that highlights changes made to the original version. You should upload this as a separate file labeled 'Revised Manuscript with Track Changes'.
- An unmarked version of your revised paper without tracked changes. You should upload this as a separate file labeled 'Manuscript'.

**Response:** Sincerely, thank you again for your efforts and contributions to our manuscript. Following your instructions:

(1) We responded positively to every point and comment and uploaded it as a separate file labeled 'Response to Reviewers'.

(2) We created a new copy of the manuscript and highlighted all the revisions in red font. This copy is uploaded as a separate file labeled 'Revised Manuscript with Track Changes'.

(3) We upload the revised manuscript as a separate file labeled 'Manuscript'. The manuscript does not contain any traces or markings.

**Comment 2**: When submitting your revision, we need you to address these additional requirements.

- Please ensure that your manuscript meets PLOS ONE's style requirements, including those for file naming. The PLOS ONE style templates can be found at

https://journals.plos.org/plosone/s/file?id=wjVg/PLOSOne_formatting_sample_main_body.pdf

https://journals.plos.org/plosone/s/file?id=ba62/PLOSOne_formatting_sample_title_authors_affiliations.pdf

- Your ethics statement should only appear in the Methods section of your manuscript. If your ethics statement is written in any section besides the Methods, please delete it from any other section.
- If the reviewer comments include a recommendation to cite specific previously published works, please review and evaluate these publications to determine whether they are relevant and should be cited. There is no requirement to cite these works unless the editor has indicated otherwise.

**Response:** Thank you for your detailed guidance.

(1) We have carefully read both the two documents and the recent publications of *PLoS One*. Based on this information, we adjusted the manuscript's style requirements as appropriate.

(2) We have removed the section of "Ethical approval" between the section of "Author contributions" and section of "Disclosure of interest". i.e.

***~~Ethical approval~~***

*~~Approval for the study was granted by the Human Institutional Review Board at Beth Israel Deaconess Medical Center. The studies were conducted in accordance with the local legislation and institutional requirements. The further ethical review was considered unnecessary for this study, as no additional data collection was undertaken. The individual patient consent was deemed unnecessary for this study, as the study was retrospective and the anonymized data was publicly available, and individuals could not be identified either directly or indirectly.~~*

(3) The comments of the two reviewers did not include suggestions to cite specific previously published works. Furthermore, under the professional guidance of editor and reviewers, the quality of manuscripts has been improved dramatically. We believe that the content of the manuscript would attract the attention of peer researchers and our team will actively cite this manuscript as a basis for further research.

**Comment 3**: We note that you have indicated that there are restrictions to data sharing for this study. For studies involving human research participant data or other sensitive data, we encourage authors to share de-identified or anonymized data. However, when data cannot be publicly shared for ethical reasons, we allow authors to make their data sets available upon request. For information on unacceptable data access restrictions, please see http://journals.plos.org/plosone/s/data-availability#loc-unacceptable-data-access-restrictions.

Before we proceed with your manuscript, please address the following prompts:

a) If there are ethical or legal restrictions on sharing a de-identified data set, please explain them in detail (e.g., data contain potentially identifying or sensitive patient information, data are owned by a third-party organization, etc.) and who has imposed them (e.g., a Research Ethics Committee or Institutional Review Board, etc.). Please also provide contact information for a data access committee, ethics committee, or other institutional body to which data requests may be sent.

b) If there are no restrictions, please upload the minimal anonymized data set necessary to replicate your study findings to a stable, public repository and provide us with the relevant URLs, DOIs, or accession numbers. Please see http://www.bmj.com/content/340/bmj.c181.long for guidelines on how to de-identify and prepare clinical data for publication. For a list of recommended repositories, please see https://journals.plos.org/plosone/s/recommended-repositories. You also have the option of uploading the data as Supporting Information files, but we would recommend depositing data directly to a data repository if possible.

Please update your Data Availability statement in the submission form accordingly.

**Response:** Thanks a lot for your guidance. We fully recognize and respect PLoS One's request for authors to share de-identified or anonymized data for research involving human study participant data or related sensitive data. This is also an important measure to improve the reliability and transparency of research and promote academic exchanges. Following your instructions, we anonymized all data from this study (without any ID number, only clinical features) and uploaded it to a stable public repository **figshare**. In addition, we have updated the Data Availability statement section in the manuscript accordingly. Line 348-350 in Revised Manuscript with Track Changes. i.e.

*"****Data availability statement***

*The datasets presented can be obtained from the following link on the premise of completing MIMIC database's training (https://doi.org/10.6084/m9.figshare.31981221)."*

**Response to Reviewer 1**

**Comment 1:** This study leverages the large sample size (n=17,624) of the MIMIC-IV database to investigate, for the first time, the value of the Endothelial Activation Stress Index (EASIX) in predicting outcomes and optimizing treatment for critically ill patients with AKI. The study design is well-structured, employing rigorous statistical methods (including Cox models, Boruta algorithm, etc.), and the conclusions hold potential clinical significance. However, certain methodological details require clarification, the presentation of results needs refinement, and the discussion section warrants greater caution. Recommended for conditional acceptance (Major Revision).

**Response:** We are very grateful for your encouraging feedback and recognition of our work, which is a great honor for our team. Under the guidance of you and other experts, We actively clarified some methodological details, improved the presentation of results, and revised some of the discussion.

**Comment 2:** The abstract and results claim that "glucocorticoid-associated risks were mitigated in patients with elevatedlog2(EASIX) levels" (p<0.001), but the main text only demonstrates this trend via the RCS curve in Figure 4D. This suggests that the 1-year mortality rate decreases in patients using glucocorticoids as log2(EASIX) increases. We recommend revising the description accordingly.

**Response:** Thank you for your detailed guidance, which indeed highlights an important aspect. Taking into account your *comment 3*, we have supplemented the HR value of glucocorticoid therapy. Based on this, we modified the description of the glucocorticoid-related risk and the risk change with log2 (EASIX) level change in the Abstract section and the Results section. Line 30-32 and 206-210 in the Revised Manuscript with Track Changes. i.e.

*"****Abstract***

***Results****: ... The glucocorticoid use may be independently associated with an increased risk of 1-year (HR: 1.27, 95% CI: 1.21-1.34) and ICU (HR: 1.39, 95% CI: 1.31-1.47) mortality.* *The glucocorticoid-associated risk decreased as the log2(EASIX) level increased (p < 0.001)."*

*"****Results***

***Subgroup analysis***

*...*

*About a quarter of patients (26.8%) of patients underwent glucocorticoid therapy. After minimizing confounding bias, this glucocorticoid use was independently associated with an increased risk of 1-year (HR: 1.27, 95% CI: 1.21-1.34) and ICU (HR: 1.39, 95% CI: 1.31-1.47) mortality (S1 Table). Meanwhile, the glucocorticoid-associated risk decreased as the log2(EASIX) level increased (p for interaction < 0.001) (Fig 4D, S1 Table)."*

**Comment 3:** There were 26.8% of patients who received glucocorticoid therapy, which may adversely affect patient prognosis (Figure 4D). We recommend adding a subgroup analysis for glucocorticoid use, supplementing with the HR values for glucocorticoids and the interaction P-values in the high/low EASIX groups, respectively.

**Response:** Thank you for your insightful guidance. Following your instructions, We have added subgroup analyses of glucocorticoid use and corresponding interaction *p*-value, which are presented in the forest plot. We have supplemented the HR value of glucocorticoid use and the interaction *p*-value of the high/low EASIX groups, which are presented in Supplementary table 1. In addition, we have revised the relevant description to take into account your *comment 2*. Line 197-199 and 206-210 in the Revised Manuscript with Track Changes. i.e.

*"****Results***

***Subgroup analysis***

*...*

*Conversely, the link was notably stronger in those experiencing mild AKI (p for interaction = 0.007), those who did not have CKD (p = 0.047), CRRT (p = 0.016), sepsis (p < 0.001) or glucocorticoid use (p < 0.001).*

*...*

*About a quarter of patients (26.8%) of patients underwent glucocorticoid therapy. After minimizing confounding bias, this glucocorticoid use was independently associated with an increased risk of 1-year (HR: 1.27, 95% CI: 1.21-1.34) and ICU (HR: 1.39, 95% CI: 1.31-1.47) mortality (S1 Table). Meanwhile, the glucocorticoid-associated risk decreased as the log2(EASIX) level increased (p for interaction < 0.001) (Fig 4D, S1 Table)."*

*"*

*Fig 3. The restricted cubic spline curve for all patients and forest plot for subgroup analysis. (A) Restricted cubic spline curve of the log2(EASIX) and hazard ratios in patients with AKI. (B) Subgroup analysis by gender, diabetes, hypertension, AKI stage, heart failure, sepsis, chronic kidney disease, glucocorticoid use, continuous renal replacement therapy, and ventilation."*

*"****S1 Table. The association between glucocorticoid use and all-cause mortality.***

| ***glucocorticoid*** | ***number*** | ***model 0*** | ***model 1*** | ***model 2*** | ***model 3*** | ***p for interaction*** |
| --- | --- | --- | --- | --- | --- | --- |
| *17624* | | ***Hazard Ratio for 1-year mortality (Cox regression)*** | | | | ***-*** |
| *No* | *12895* | *reference* | *reference* | *reference* | *reference* |  |
| *Yes* | *4729* | *1.54 (1.47, 1.61)* | *1.47 (1.40, 1.54)* | *1.29 (1.23, 1.36)* | *1.27 (1.21, 1.34)* |  |
| *11346* | | *Only in patients with low-level log2(EASIX)* | | | | *< 0.001* |
| *No* | *8635* | *reference* | *reference* | *reference* | *reference* |  |
| *Yes* | *2711* | *1.57 (1.47, 1.67)* | *1.54 (1.45, 1.65)* | *1.42 (1.33, 1.52)* | *1.42 (1.32, 1.51)* |  |
| *6278* | | *Only in patients with high-level log2(EASIX)* | | | |  |
| *No* | *4260* | *reference* | *reference* | *reference* | *reference* |  |
| *Yes* | *2018* | *1.34 (1.25, 1.44)* | *1.32 (1.23, 1.42)* | *1.12 (1.05, 1.21)* | *1.10 (1.02, 1.18)* |  |
| *17624* | | ***Relative Risk for ICU mortality (modified Poisson regression)*** | | | | ***-*** |
| *No* | *12895* | *reference* | *reference* | *reference* | *reference* |  |
| *Yes* | *4729* | *1.88 (1.77, 1.99)* | *1.65 (1.56, 1.75)* | *1.42 (1.34, 1.51)* | *1.39 (1.31, 1.47)* |  |
| *11346* | | *Only in patients with low-level log2(EASIX)* | | | | *< 0.001* |
| *No* | *8635* | *reference* | *reference* | *reference* | *reference* |  |
| *Yes* | *2711* | *1.92 (1.75, 2.11)* | *1.73 (1.58, 1.90)* | *1.50 (1.37, 1.65)* | *1.48 (1.34, 1.62)* |  |
| *6278* | | *Only in patients with high-level log2(EASIX)* | | | |  |
| *No* | *4260* | *reference* | *reference* | *reference* | *reference* |  |
| *Yes* | *2018* | *1.62 (1.50, 1.74)* | *1.50 (1.39, 1.61)* | *1.30 (1.21, 1.40)* | *1.28 (1.18, 1.37)* |  |

*model 0: only glucocorticoid use was included.*

*model 1: age, gender, diabetes, hypertension, AKI stage, CKD, heart failure, cirrhosis, sepsis, and cancer were adjusted.*

*model 2: age, gender, diabetes, hypertension, AKI stage, CKD, heart failure, cirrhosis, sepsis, and cancer, as well as WBC, hemoglobin, RDW, platelets, AST, ALT, TBil, albumin, creatinine, BUN, LDH, lactate, anion gap, antibiotic, RAASi, ventilation, and CRRT were adjusted.*

*model 3: age, gender, diabetes, hypertension, AKI stage, CKD, heart failure, cirrhosis, sepsis, cancer, WBC, hemoglobin, RDW, platelets, AST, ALT, TBil, albumin, creatinine, BUN, LDH, lactate, anion gap, antibiotic, RAASi, ventilation, and CRRT, as well as SOFA and APS Ⅲ were further adjusted."*

**Comment 4:** It is recommended to include a discussion and analysis of the potential mechanisms underlying the reduction in mortality associated with glucocorticoid use in high-level EASIX groups.

**Response:** We thank the reviewer for this important consideration. In the fourth paragraph of the Discussion section, we have discussed the associated content of the reduction in mortality associated with glucocorticoid use in the high-level EASIX group. However, it did not include the underlying mechanism. Following your instructions, we have revised some expressions and expanded the Discussion in this section to include an analysis of potential mechanisms. Line 284-307 in the Revised Manuscript with Track Changes. i.e.

*"****Discussion***

*...*

*In cases of sepsis-induced AKI, glucocorticoid use, particularly with hydrocortisone, has been observed to inhibit pro-inflammatory cytokines and to enhance serum creatinine levels and urine output, indicating potential improvements in renal function[32]. Nevertheless, there is increasing concern regarding the safety of glucocorticoids in patients with AKI, especially concerning their long-term effects. While acute administration may mitigate inflammation and improve survival in critical situations, prolonged glucocorticoid use may result in metabolic adverse effects, potentially complicating patient outcomes[33]. During the COVID-19 pandemic, the use of high-dose glucocorticoids was linked to a heightened risk of severe complications in patients with pre-existing kidney disease[34]. Previous exposure to glucocorticoids may be associated with an increased mortality and a heightened risk of complications related to AKI[35]. Our analysis indicated that glucocorticoid administration may have adverse effects on this patient cohort. However, these effects appeared to be mitigated in individuals with elevated EASIX levels. The underlying mechanism may lie in the targeted reversal of the pathological state of endothelial by glucocorticoids. Studies have shown that glucocorticoids could enhance endothelial barrier function by activating the SphK1-S1P signaling pathway and inhibit the expression of adhesion molecules such as ICAM-1 by inhibiting the NF-κB pathway, thereby reducing vascular leakage and inflammatory storms[36,37]. High EASIX may indicate impaired endothelial barrier integrity and microcirculatory disturbances, and the protective effects of glucocorticoids could counteract this core pathophysiological process, thereby counteracting the potential side effects of the drug, manifested by a decrease in risk. On the contrary, patients in the low EASIX group had milder endothelial stress and a narrow window for glucocorticoid treatment, and their non-specific immunosuppressive and metabolic side effects may outweigh the benefits of endothelial protection, leading to increased risk. Based on this, the modulation of therapeutic outcomes by EASIX could facilitate the refinement and optimization of EASIX-guided treatment strategies. ..."*

**Comment 5:** It is recommended that figures and charts in the manuscript be standardized according to journal requirements, including but not limited to units, abbreviation explanations, and numerical formats. Image quality needs improvement.

**Response:** Thanks for the meticulous advice. Following your and the editor's guidance, we have standardized the format and content of the manuscript in accordance with the journal's requirements. In addition, our Images are all 300dpi resolution. In the submission system - PDF file, the image is compressed. In the PDF file used for peer review, a download link to the original image is provided in the upper right corner of the Figure page. Again, we would like to express our sincere gratitude for your valuable efforts and professional evaluation.

**Response to Reviewer 2**

**Comment 1:** I have read the paper with great interest. This study provides a rigorous and well-executed retrospective analysis of the endothelial activation and stress index in critically ill patients with acute kidney injury using the MIMIC-IV database. The methodology is robust, incorporating advanced statistical techniques such as overlap-weighted propensity scores, restricted cubic splines, and the Boruta algorithm. The findings are clinically relevant and offer valuable insights into risk stratification and potential treatment optimization. Overall, my impression of this manuscript is highly positive, and I believe it is a strong candidate for publication after minor revisions.

**Response:** Thank you very much for your comprehensive evaluation of our manuscript and for your encouraging comments on our team's work. Under the guidance of you experts, we actively completed the revision of the manuscript. Our team has always been committed to scientific research close to the clinic and hopes to continue to maintain this academic style for more excellence.

**Comment 2:** Please standardize the database name throughout (e.g., MIMIC-Ⅳ), avoiding mixed forms such as "MIMIC - IV," "MIMIC - Ⅳ," etc. The Methods state "from 2008 and 2022," which should read "from 2008 to 2022".

**Response:** Thank you for your detailed feedback. Following your instructions, we revised the relevant text from start to finish. They include, but are not limited to "MIMIC-Ⅳ", and "from 2008 to 2022".

**Comment 3:** EASIX is defined using the first measurement within 24 hours of ICU admission, but some patients may have already developed AKI prior to ICU admission. Please clarify in the Methods the exact AKI identification/ascertainment window and how this aligns temporally with the exposure definition, to reduce concerns about reverse causality.

**Response:** Thank you for the professional review. This is an especially important issue. We have defined AKI based on changes in serum creatinine level and urine output after ICU admission. Indeed, we should clarify this sufficiently to reduce related concerns. In the revised manuscript, we have added this to the explanation. Line 88-90 in the Revised Manuscript with Track Changes. i.e.

*"****Materials & Methods***

***Study population***

*...*

*Patients with AKI as a first-time ICU admission were enrolled. Specifically, AKI was diagnosed according to the KDIGO guidelines based on the level of serum creatinine and urine output after ICU admission, but not according to the International Classification of Diseases[20,21]. "*

**Comment 4:** The manuscript uses "relative ratio" in places, please consider standardizing to relative risk (RR) where modified Poisson regression is used.

**Response:** Thank you for your detailed comment. In the revised manuscript, we have modified the corresponding vocabulary to achieve a more appropriate wording. Line 125-127 in the Revised Manuscript with Track Changes. i.e.

*"****Statistical analysis***

*...*

*Modified Poisson regression was used to calculate the relative risk (RR) for log2(EASIX), considering both ICU mortality and 1-year mortality as outcomes to ensure the analysis reliability."*

**Comment 5:** Please standardize units/capitalization (e.g., mg/dL rather than mg/dl) in Table 1.

**Response:** Thank you for your detailed guidance. In the revised manuscript, we have revised the corresponding vocabulary. Line 162 in the Revised Manuscript with Track Changes. i.e.

*"****Table 1. Baseline information according to the levels of log2(EASIX).***

*...*

*total bilirubin (mg/dL)*

*creatinine (mg/dL)*

*blood urea nitrogen (mg/dL)"*

**Comment 6:** There is a very minor inconsistency in the spelling of "Poisson" regression in Table 2 (currently written as "Poison regression"). This is a trivial typographical error that should be corrected.

**Response:** Thank you for your detailed feedback. In the revised manuscript, we corrected this incorrect wording. Line 176 in the Revised Manuscript with Track Changes. i.e.

*"****Table 2. The association between log2(EASIX) and all-cause mortality.***

*...*

*Relative Risk for ICU mortality (modified Poisson regression)*

*Relative Risk for 1-year mortality (modified Poisson regression)"*

**Comment 7:** In Table 2, the hazard ratios (HRs) for "Each unit increase" show a pattern of initially decreasing and then increasing across different models (model1 1.14，model2 1.20，model3 1.17). I would suggest that the authors briefly explain this in the Table note to avoid confusion for readers.

**Response:** Thank you for your detailed feedback and this is an important clarification request.

Thanks for the meticulous advice. As you mentioned, this could cause misunderstandings among readers. In the text of Results section, we have revised the relevant description. In Table 2, we have described more clearly the confounding factor control in different regression models. Hopefully, these changes would respond to this issue. Line 169-172 and 177-185 in the Revised Manuscript with Track Changes. i.e.

*"****Results***

***Risk ratios for all-cause mortality***

*...*

*In model 3, elevated log2(EASIX) were linked to an increased mortality (HR: 1.41, 95% CI: 1.32-1.50), and a unit increase in log2(EASIX) was linked to a 17% higher risk. Consistent findings were observed across models 0, 1, and 2 (Table 2)."*

*"****Table 2. The association between log2(EASIX) and all-cause mortality.***

*...*

*model 0: only log2(EASIX) was included.*

*model 1: age, gender, diabetes, hypertension, AKI stage, CKD, heart failure, cirrhosis, sepsis, and cancer were adjusted.*

*model 2: age, gender, diabetes, hypertension, AKI stage, CKD, heart failure, cirrhosis, sepsis, and cancer, as well as WBC, hemoglobin, RDW, platelets, AST, ALT, TBil, albumin, creatinine, BUN, LDH, lactate, anion gap, glucocorticoid, antibiotic, RAASi, ventilation, and CRRT were adjusted.*

*model 3: age, gender, diabetes, hypertension, AKI stage, CKD, heart failure, cirrhosis, sepsis, cancer, WBC, hemoglobin, RDW, platelets, AST, ALT, TBil, albumin, creatinine, BUN, LDH, lactate, anion gap, antibiotic, RAASi, ventilation, and CRRT, as well as SOFA and APS Ⅲ were adjusted."*

**Comment 8:** In the figure legend the manuscript uses "shaded feature," whereas the main text alternates between "shadow feature" and "shaded feature." Since the standard Boruta terminology is "shadow features," please unify the wording throughout () to avoid confusion.

**Response:** Thank you for your insightful guidance. Following your instructions, we have re-correctly used the term "shadow features" in text, legends, and any supplementary materials. Again, thank you for your careful comments, this rigorous attitude would deeply affect our subsequent research work.
